# Thermal and Nonthermal Effects of 5 G Radio-Waves on Human's Tissue

**DOI:** 10.1155/2024/3801604

**Published:** 2024-07-24

**Authors:** Yahia Hasan Jazyah

**Affiliations:** Faculty of Computer Studies Arab Open University, Al-Ardiya, Kuwait

## Abstract

The deployment of 5 G wireless technology has generated considerable interest and debate regarding its potential effects on human health. This work provides a comprehensive overview of the current scientific understanding of the potential health implications associated with 5 G technology. Drawing upon a wide range of research studies, reviews, and expert opinions, we explore the implications through which 5 G signals interact with the human body. This work presents a balanced perspective, summarizing both the potential benefits of 5 G technology, such as improved data transfer speeds, reduced latency, and enhanced connectivity, as well as concerns that have been raised about its effects on human's tissues. We discuss various aspects of health impacts, including thermal and nonthermal effects, focusing on the existing research on radiofrequency electromagnetic fields and their potential to cause adverse health outcomes. Simulation results show the negative effect of radio waves on human's tissues.

## 1. Introduction

The rapid advancement of wireless communication technologies has revolutionized the way we connect, communicate, and access information. 5 G wireless technology stands at the forefront of this digital transformation, promising high data speeds, reduced latency, and enhanced connectivity. As 5 G networks continue to be deployed worldwide, they hold the potential to reshape industries, support the Internet of Things (IoT), and enable novel applications that were previously unimaginable. However, alongside the excitement and anticipation surrounding 5 G's capabilities, concerns about its potential impact on human health have also emerged.

The interaction between electromagnetic field (EMF) and the human body has been a subject of scientific inquiry for decades, and the rollout of each new wireless generation prompts renewed examination. With 5 G technology utilizing higher-frequency bands, including millimeter waves (mmWave), questions have been raised about whether these new frequencies and increased exposure levels could have implications for human well-being. While extensive research has been conducted to assess the potential health effects of radiofrequency radiation, the advent of 5 G has introduced a new dimension to this discourse [1].

This paper provides a comprehensive exploration of the impact of 5 G technology on human health. It aims at providing a balanced overview of the current state of knowledge by investigating into both the potential benefits that 5 G offers and the existing concerns surrounding its effects. The discussion encompasses various dimensions, including thermal and nonthermal effects; in addition, it provides a study about a real scenario and calculates the received power that human can bear in different high-frequency scenarios.

Simulation results show that the higher frequency, the higher the effect on the human body.

The remaining part of this article is organized as follows: part 2 presents an overview about 5 G, part 3 presents the safety of 5 G technology, part 4 presents the literature review, part 5 presents thermal and nonthermal effects, part 6 presents the relation between Specific Absorption Rate (SAR) and distance, part 7 presents the relationship between SAR and nonthermal effect, part 8 presents the results and analysis, and part 9 is the conclusion.

## 2. 5 G Technology

### 2.1. Definition

5 G wireless technology [2] represents the latest evolution in mobile communication and networking standards. It builds upon the foundation established by its predecessors, such as 3 G and 4 G/LTE, with the primary goal of providing significantly enhanced data rate, low latency, and better connectivity. 5 G technology is designed to meet the growing demands of modern communication and to support emerging technologies such as the IoT, autonomous vehicles, augmented reality, and virtual reality.

mmWave is regarded as wavelengths from 1 to 10 mm and frequencies from 30 to 300 GHz, and the current 5 G communications include frequencies below 30 GHz, such as the 28-GHz band, and upper limit frequencies up to 100 GHz [2].

As with any technological advancement, the deployment of 5 G has sparked discussions and concerns about its potential impact on various aspects of society, including health and safety. Ongoing research and regulatory measures aim to ensure that the benefits of 5 G are maximized while potential risks are adequately managed.

### 2.2. Benefits

The deployment of 5 G wireless technology promises a new era of connectivity and innovation, promising a great of benefits that have the potential to transform industries, enhance user experiences, and enable novel applications [2].

Not only does 5 G enhance data rate and latency, but also it provides massive device connectivity, enhances reliability, improves energy efficiency, and increases network capacity.

Moreover, it enables augmented reality (AR) and virtual reality (VR) to drive economic growth and innovation through the creation of new markets, services, and business models. Revolutionizes healthcare by enabling real-time telemedicine consultations, remote diagnostics, and even remote surgeries. Develop smart cities by providing sustainable infrastructure and reducing energy consumption. Improves public safety and emergency response through faster and more reliable communication [3].

### 2.3. Major Issues

The adoption of 5 G wireless technology has introduced several significant issues and challenges that need to be addressed to ensure that the technology is deployed safely and effectively [4].

One of the most debated issues surrounding 5 G is its potential impact on human health. While extensive research has been conducted on the safety of radiofrequency electromagnetic fields (RF-EMF) [5], concerns have been raised about the higher-frequency mmWaves used in 5 G. Some studies suggest possible thermal and nonthermal effects on human tissues and cells, but the consensus among regulatory bodies is that 5 G technology is safe as long as exposure remains within established guidelines. This will be discussed later in detail.

5 G networks require a significant infrastructure upgrade due to the use of higher-frequency bands and smaller cell sizes. This includes the installation of a larger number of smaller cell stations and distributed antenna systems. This in turn, reflects on the cost, and regulatory approvals [6].

The proliferation of a large number of connected devices and the massive data flows generated by 5 G networks raise concerns about privacy and data security and enable the potential for cyberattacks and unauthorized access.

5 G requires efficient access to a range of frequency bands, that can lead to competition for the limited spectrum resources and cause interference between different services and technologies.

5 G is designed to be more energy-efficient than previous generations; nevertheless, the increased number of connected devices and infrastructure components could lead to higher energy consumption. This leads to a challenge of balancing between the benefits of 5 G with the need for energy conservation and sustainability remains [7].

The rapid evolution of 5 G technology outpaces regulatory and policy development. Governments and regulatory bodies must adapt their frameworks to address the challenges raised by 5 G, including spectrum allocation, infrastructure deployment, privacy, and safety standards [8].

International cooperation and standardization are required due to the global nature of 5 G technology to ensure interoperability and seamless communication across different networks and regions.

5 G technology offers transformative capabilities, but it is accompanied by a range of challenges that need to be carefully managed.

### 2.4. 5 G and Former Counterparts

Each mobile generation builds upon the previous generation, with 5 G representing the latest advancement in wireless communication technology, offering significantly faster data speeds, lower latency, and support for a broader range of applications.  Table 1 provides a comparison of the major aspects of each mobile generation, including data transfer speeds, technology, frequency bands, spectrum efficiency, and latency [2].

## 3. 5 G Safety

### 3.1. Is 5 G Safer than Wi-Fi?

**Figure 1:**
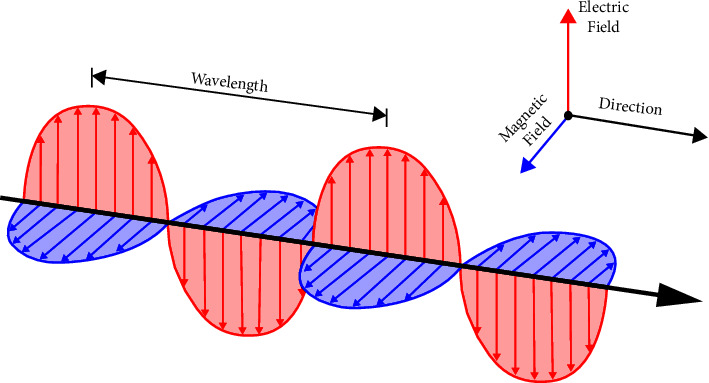
Electromagnetic wave (radio wave).

Both 5 G and Wi-Fi are wireless communication technologies, but they operate differently and serve distinct purposes. Whether 5 G is safer than Wi-Fi or not in terms of health effects depends on electromagnetic radiation exposure and the potential associated risks. To understand this point, it is necessary to understand what electromagnetic radiation and exposure levels are. Radio Wave or electromagnetic wave is the signal that consists of two components, magnetic field and electric field, both of which are perpendicular to each other, and they are moving in a direction perpendicular to them, see Figure 1. [9].

Wi-Fi [10] operates on radiofrequency (RF) electromagnetic fields, typically in the frequency bands of 2.4 and 5 GHz. Those frequencies are lower than the frequencies used in some 5 G deployments.

mmWave [1] is regarded as wavelengths from 1 to 10 mm and frequencies from 30 to 300 GHz, but current 5 G communications include frequencies below 30 GHz, such as the 28-GHz band, and frequencies up to about 100 GHz as the upper limit.

Wi-Fi [10] signals are generally lower in power and are used for shorter-range communication such as homes, offices, and public places. While 5 G networks use a combination of small and macrocells to provide coverage. Higher frequency mmWave 5 G signals have a shorter range and are often used in densely populated urban areas for improved capacity and speeds.

Both 5 G and Wi-Fi emit nonionizing radiation, which means they do not have enough energy to ionize atoms or molecules and cause damage at the cellular level, while ionizing radiation such as X-rays and nuclear radiation have enough energy to ionize atoms or molecules.

The potential health effects of exposure to nonionizing radiation, including RF-EMF, have been studied for years. The consensus among international health organizations (such as the World Health Organization and the International Commission on NonIonizing Radiation Protection) is that there is currently no conclusive evidence that RF-EMF generated by Wi-Fi or 5 G raises significant health risks when exposure remains within established safety guidelines [11].

Both Wi-Fi and 5 G technologies are subject to safety guidelines and regulations set by various national and international bodies. Which set exposure limits to ensure that the levels of electromagnetic radiation emitted by devices and infrastructure are below the levels that can affect health negatively. More details are in next sections.

### 3.2. 5 G and Air Safety

The deployment of 5 G technology has raised concerns about its potential impact on air safety. [12].

One of the primary concerns is the potential for radio frequency interference between 5 G signals and the communication, navigation, and surveillance systems used in aviation. Those systems depend on specific frequency bands to ensure accurate and reliable aircraft communication and navigation. If 5 G signals interfere with these bands, it could lead to disruptions in communication between air traffic control and aircraft or affect the accuracy of navigation systems.

Aviation systems [13], such as Instrument Landing Systems (ILS) and radar altimeters, are crucial for safe landing approaches and altitude measurements. These systems operate in frequency bands that are adjacent to those used for 5 G. Interference from 5 G signals could compromise the integrity and reliability of these critical aviation systems.

Regulatory authorities, such as the Federal Aviation Administration (FAA) in the United States and the European Union Aviation Safety Agency (EASA), have expressed concerns about potential interference with aviation systems. They require testing and mitigation measures before allowing the deployment of 5 G infrastructure near airports.

## 4. Literature Review

Belyaev [14] provides an overview of the complex dependence of the nonthermal microwave effects on various physical and biological variables, and it proved that the health effects of chronic MMW exposures may be more significant than for any other frequency range. Mandl et al. [15] present two different measurement setups to study the effect of electromagnetic radiation on human's body, results of measurements compared with legal and health limits, The calculated legal exposure limits of mobile devices were exceeded twice in areas within very poor reception values. Fernández et al. [16] present an evaluation of electromagnetic exposure due to 5 G mobile communications in outdoor environments, comparison with exposure limits and with the results derived from a measurement campaign was carried out. Sofri et al. [17] present a review and analyses of the latest research on electromagnetic exposure on humans, with particular attention to its effect on cognitive performance, well-being, physiological parameters. Thotahewa et al. [18] presented the electromagnetic effects of head implantable transmitting devices operating based on Impulse Radio Ultra-Wide Band (IR-UWB) wireless technology, it was observed that SAR is highly dependent on the bandwidth of the IR-UWB signal. Alcaras and Frere [19] present a new concept of heterogeneous phantoms to enhance SAR method validation of radio military exposure near high power antennas. Laissaoui et al. [20] present a dosimetrist study by calculating the SAR and the temperature in the human head exposed to electromagnetic radiation emitted by a Smartphone. Nasiar and Rani in [21] discuss the dangers of the mobile phone radiation on human health. Annalakshmi and Umarani [22] investigated human EMF exposure from 5 G wireless communication. It analyses the impact of the penetration level of EMF in human skin at 28 GHz, it shows that SAR levels vary depending on several key factors, including frequency and material type. Di Ciaula [23] provides a huge review about the impact of 5 G radio waves on human health, and the conclusion indicates that there is a negative effect of radio waves on health. Simkó and Mattsson in [24] analyse 94 relevant publications performing in vivo or in vitro investigations. It concludes that the available studies do not provide adequate and sufficient information for a meaningful safety assessment, or nonthermal effects. Also suggests research regarding local heat developments on small surfaces of the human body.

Bozorgmanesh et al. in [25] conducted an interference analysis to investigate the compatibility between 5 G wireless systems and radar altimeters, and a strategy was proposed to mitigate interference by reducing the transmission power of 5 G Base Stations (BSs) in certain situations or specific geographic areas. Abdul-Al et al. in [26] investigate the potential interference between 5 G New Radio (NR) and radar altimeters in various environments, and their simulation results revealed significant interference effects on radar altimeters from various 5 G BS configurations. Yang et al. in [27] reviews and quantifies flight hazards from 5 G interference. Graesslin et al. in [28] identify the requirements of 5 G implementation in various airport use cases and propose detailed 5 G solutions for these cases. Li et al. in [29] investigated the impact of different flight altitudes and horizontal isolation distances on system performance in urban and rural areas, simulation results show that there exists a compatibility issue between the 5 G system and radio altimeters in the 4400–4500 MHz band.

Table 2 summarises the literature about the effects of thermal and nonthermal effects on human's tissues in addition to their effects on aircraft safety.

## 5. Thermal and Nonthermal Effects

### 5.1. Thermal Effects

Thermal effects [30] are related to the ability of electromagnetic radiation (including radio waves used in wireless communication, such as 5 G) to generate heat in biological tissues due to the absorption of energy from the radiation by the tissue. The energy absorbed can lead to a rise in temperature, potentially affecting cellular processes. The effect of such heat could occur when the power levels of the radiation are relatively high.

Regulatory agencies around the world, such as the Federal Communications Commission (FCC)—the United States, set exposure limits for electromagnetic radiation, including the wireless communication technologies, in order to ensure that the levels of radiation are below what is known to cause significant thermal effects that can affect human health negatively.

The thermal effect of radio waves can be described by the Specific Absorption Rate [31], which is a measure of the rate at which power is absorbed (P) per unit mass (M) of the material or tissue being exposed to electromagnetic radiation, measured in watts per kilogram (W/kg), as shown in the following equation:

(1) $$\begin{array}{c} SAR=\frac{P}{M}. \end{array}$$

Equation (1) represents simple and general formula to calculate SAR.

Going into a more detailed formula of SAR, the calculation of the thermal effect includes various factors. As shown in the following equation [31]:

(2) $$\begin{array}{c} SAR=\frac{{\sigma E}^{2}}{\rho }, \end{array}$$

where *σ* is the electrical conductivity of the tissue, *E* is the electric field strength of the radio wave, and *ρ* is the density of the tissue.

The electric field strength can be calculated by the following equation [32]:

(3) $$\begin{array}{c} E=\frac{\sqrt{S}}{2}, \end{array}$$

where *i* is the power density of the radio wave and *Z*_0_ is the characteristic impedance of free space (120*π* Ω).

Equation (4) presents more details that consider additional factors for calculating the specific absorption rate due to radio wave exposure, considering tissue properties and frequency-dependent effects [33].

(4) $$\begin{array}{c} SAR=\frac{2\pi \epsilon^{\prime \prime }\left(f\right){\left|E\left(f\right)\right|}^{2}}{\rho } \end{array}$$

where *f* is the frequency of the radio wave, *ϵ*^″^(*f*) is the frequency-dependent dielectric loss factor of the tissue, and |*E*(*f*)|^2^ is the square of the electric field amplitude of the radio wave.

The dielectric loss factor *ϵ*^″^(*f*) [34] is the energy dissipation due to the dielectric properties of the tissue. It's a complex quantity that represents the imaginary part of the relative permittivity (*ϵ*_*r*_) of the tissue. The real and imaginary parts of the relative permittivity are given by the following equation:

(5) $$\begin{array}{c} \epsilon_{r}\left(f\right)=\epsilon^{\prime }+j\epsilon^{\prime \prime }\left(f\right), \end{array}$$

where *ϵ*′ is the real part of the relative permittivity, and *j* is the imaginary unit.

Regulatory agencies set SAR limits to ensure that the energy absorbed by the human body from radio waves does not lead to significant temperature increases that could harm human health.

The implementation of 5 G technology has sparked interest and debate regarding its potential effects on human health which include [24, 35–37].

At higher frequencies used in certain 5 G deployments, RF-EMF absorption by the body may result in localized tissue heating. However, the extent of this heating is typically limited to the skin and superficial tissues due to the short penetration depth of mmWaves.

SAR measures the rate at which RF energy is absorbed by the body and is used to assess potential thermal effects. Regulatory agencies such as the International Commission on Nonionizing Radiation Protection (ICNIRP) have established SAR limits to ensure that 5 G devices comply with safety guidelines and do not exceed permissible exposure levels.

To minimize potential thermal effects, 5 G infrastructure and devices are designed to comply with SAR limits and safety standards. Additionally, regulatory bodies monitor and enforce compliance with these standards to protect public health.

### 5.2. Nonthermal Effects

Nonthermal effect [38] refers to potential biological effects of electromagnetic radiation that do not involve a significant rise in temperature. It is generally much weaker and more subtle than thermal effects. This effect is the subject of ongoing research and debate within the scientific community.

Some studies have suggested that nonthermal effect might occur at lower levels of exposure to electromagnetic radiation. It could involve alterations in cellular functions, gene expression, and other biological responses. However, the scientific consensus until this moment is that the evidence for significant nonthermal effects from radio frequency including that emitted by 5 G technology, is limited and inconclusive.

A general representation of the relationship between nonthermal effects and radio wave exposure can be expressed by the following equation:

(6) $$\begin{array}{c} \text{Biological Response}=f\left(\left|E\right|,f,t,\text{Biological Factors}\right), \end{array}$$

where Biological Response represents the observed biological effect or response to radio wave exposure, |*E*| is the magnitude of the electric field strength of the radio wave, *f* is the frequency of the radio wave, and *t* is the duration of exposure.

Biological Factors [37] includes any relevant biological or physiological parameters that might influence the response, such as cell type, genetic factors, receptor interactions, and signalling pathways.

Equation (7) presents more detailed information about measuring the biological response [39]:

(7) $$\begin{array}{c} \text{Biological Response}=\underset{i}{\sum }\left(\text{Interactio}n_{i}\right)\left(\text{Biological Factors}\_i\right), \end{array}$$

where Interaction_*i* represents a specific interaction between electromagnetic fields and biological molecules or processes, and Biological Factor_*i* represents the influence of the specific interaction on the overall biological response.

Examples of potential interactions (Interaction_*i*) that contribute to nonthermal effects include Membrane Receptors, Ion Channels, Gene Expression, Reactive Oxygen Species (ROS), and Heat Shock Proteins (HSPs) [40, 41].

To study nonthermal effects more comprehensively, researchers often use experimental techniques and computational models to analyse changes in cellular processes, gene expression, protein activity, and other biomolecular interactions.

One commonly used empirical model to describe the probability of a biological response due to nonthermal effects is the Hill equation (equation (8)). [42].

(8) $$\begin{array}{c} \text{Response}=\frac{1}{1+{\left(EC_{50}/D\right)}^{n}}, \end{array}$$

where EC_50_ is the half-maximal effective concentration, a measure of the radiation level at which the response is half-maximal, *D* is the dose of the radiation (e.g., electric field strength and power density), and *n* is the Hill coefficient, describing the steepness of the response curve.

Regulatory agencies establish safety guidelines based on the potential for both effects that are intended to ensure that the levels of electromagnetic radiation to which the public is exposed remain below the critical threshold.

The implementation of 5 G technology has sparked interest and debate regarding its potential effects on human health which include: [14, 36, 43].

Exposure to RF-EMF may elicit biological responses in cells and tissues beyond mere tissue heating. These nonthermal effects may include alterations in cellular signalling pathways, gene expression patterns, and oxidative stress levels.

There is an impact of RF-EMF exposure on the nervous system, including effects on neuronal activity, neurotransmitter release, and blood-brain barrier permeability. There are links between RF radiation and neurological disorders such as Alzheimer's disease and Parkinson's disease.

The effects of RF-EMF exposure on reproductive health have raised concerns about potential impacts on sperm quality, fertility, and pregnancy outcomes. Animal studies have shown mixed results.

The association between RF-EMF exposure, particularly from wireless communication devices, and cancer risk remains a topic of debate. While some studies have reported increased cancer incidence in populations with high RF exposure, others have found no significant correlation. The International Agency for Research on Cancer (IARC) has classified RF radiation as a Group 2B “possible carcinogen” based on limited evidence in humans and inadequate evidence in animals.

There are potential psychological and behavioral effects of RF-EMF exposure, including changes in mood, cognition, and sleep patterns. However, the evidence in this area is limited and inconclusive.

## 6. SAR vs Distance

SAR and distance [31] from a radiation source are influenced by multiple factors, including the inverse square law, antenna characteristics, and tissue properties, as shown in the following equation:

(9) $$\begin{array}{c} SAR=\frac{S.4\pi r^{2}.t}{V\cdot \rho }, \end{array}$$

where *r* is the distance from the source, *V* is the volume of tissue exposed, and *S* is the power density given in the following equation:

(10) $$\begin{array}{c} S=\frac{P}{4\pi r^{2}}. \end{array}$$

Equations (9) and (10) show the relation between the SAR and distance from the source of power; as the distance decreases, the SAR increases.

**Figure 2:**
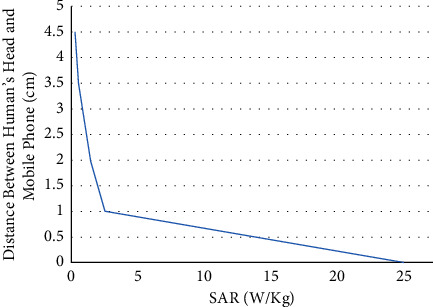
Distance vs SAR.

Assuming a transmission power of 1.9 W, frequency of 3.3 GHz, the relationship between SAR and distance is represented in Figure 2.

It is clear that when the mobile phone is very close to the human's ear, SAR is very high, and so it is recommended to use handsfree instead, knowing that the maximum limit of SAR for mobile phones based on FCC is 1.6 W/kg averaged over 1 gram of tissue. While The European Union sets the limit to 2.0 W/kg averaged over 10 grams of tissue.

The power received by the mobile phone is given by the following equation:

(11) $$\begin{array}{c} P_{r}=p_{t}-p_{l}, \end{array}$$

where Pr is the received power (dB), Pt is the transmitted power (dB), and *P*_*l*_ is the free space path loss (dB).

The free space path loss is given in equation (12) [44].

(12) $$\begin{array}{c} p_{l}=20\;\log_{10}\;\left(\frac{4\pi d}{\lambda }\right), \end{array}$$

where *d* is the distance between the mobile device and BS and *λ* is the signal wavelength.

*λ* is calculated by the following equation:

(13) $$\begin{array}{c} \lambda =\frac{c}{f}, \end{array}$$

where *c* is the speed of light (3*e*8 m/s).

When transmitted power by BS (dB) = 30, frequency = 1 MHz, distance = 100 m, and then, Free Space Path Loss is 12.44 dB, and received power: 17.56 dB.

The limited power by humans is 55 dB based on FCC records. Increasing the frequency up to 28 GHz will result in a Free Space Path Loss of 101.38 dB and received power of 71.38 dB (when *d* = 100 m) [45].

The bad impact of radio waves of the 5 G system increases when humans are close to the source of signal.

It is worth noting that EuroNews mentioned in its report that the electromagnetic radiation from iPhone 12 mobiles reached to 5.74 W/kg which exceeded the limit of 4 (W/kg) and made the French authorities to withdraw iPhone 12 from sale. France's National Frequency Agency (ANFR) ordered Apple to implement all available means to quickly remedy this malfunction [46].

## 7. SAR vs Nonthermal Effect

It is difficult to link directly between SAR and nonthermal effects associated with exposure to radiofrequency generated by 5 G networks for example, because nonthermal effects are more challenging to quantify and model compared to thermal effects, as they may involve complex cellular and molecular interactions.

Equation (14) represents the preliminary nonthermal effect in terms of SAR [41].

(14) $$\begin{array}{c} \text{Nonthermal effect}=\beta .h.i.SAR, \end{array}$$

where *β* is a proportionality constant, *h* is biological factors, and *i* is exposure characteristics.

The value of *β* is determined empirically through experimental data or theoretical considerations. It could vary depending on the specific biological system, exposure conditions, and other relevant factors. It reflects the specific conditions and context of the relationship between the variables in equation (14).

## 8. Results and Analysis

This section calculates the SAR of different body tissues over different frequencies.  Table 3 presents the values of dielectric properties (conductivity and relative permittivity) of the human's body at different frequencies.

Using equations (4) and (5), Table 4 shows SAR values for different human tissues whe the distance between antenna and human body is 0 m (worst case scenario) and peak power is 120 mW.

It is noticed from the calculated values of SAR in Table 4 that the values of SAR at 5.6 GHz are higher than the safety limit by FCC due to the high conductivity of the tissues, where conductivity is directly proportional to frequency.

**Figure 3:**
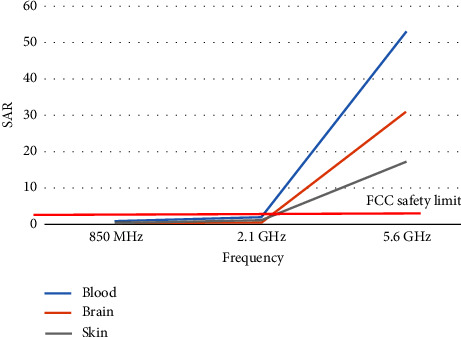
Frequency vs SAR.

Figure 3 presents the relation between SAR and frequency when the distance between the human body and antenna is 0, and the value of SAR increases when the frequency increases.

Next, equation (15) represents the temperature at a given time of body's tissue with relation to SAR [49].

(15) $$\begin{array}{c} T\left(t\right)=T_{b}+\frac{SAR}{\rho_{b}c_{b}\omega }\left(1-e^{\frac{\rho_{b}c_{b}\omega t}{c}}\;\right), \end{array}$$

where *T*_*b*_ is the body temperature of 36.6°C, *ρ*_*b*_ is the blood mass density (kg/m^3^), *c*_*b*_ is blood heat capacity (J/(kg/C)), *ω* is blood perfusion rate (ml/g/min), *c* is heating capacity of tissue (J/(kg/°C)), and *t* is the time.

**Figure 4:**
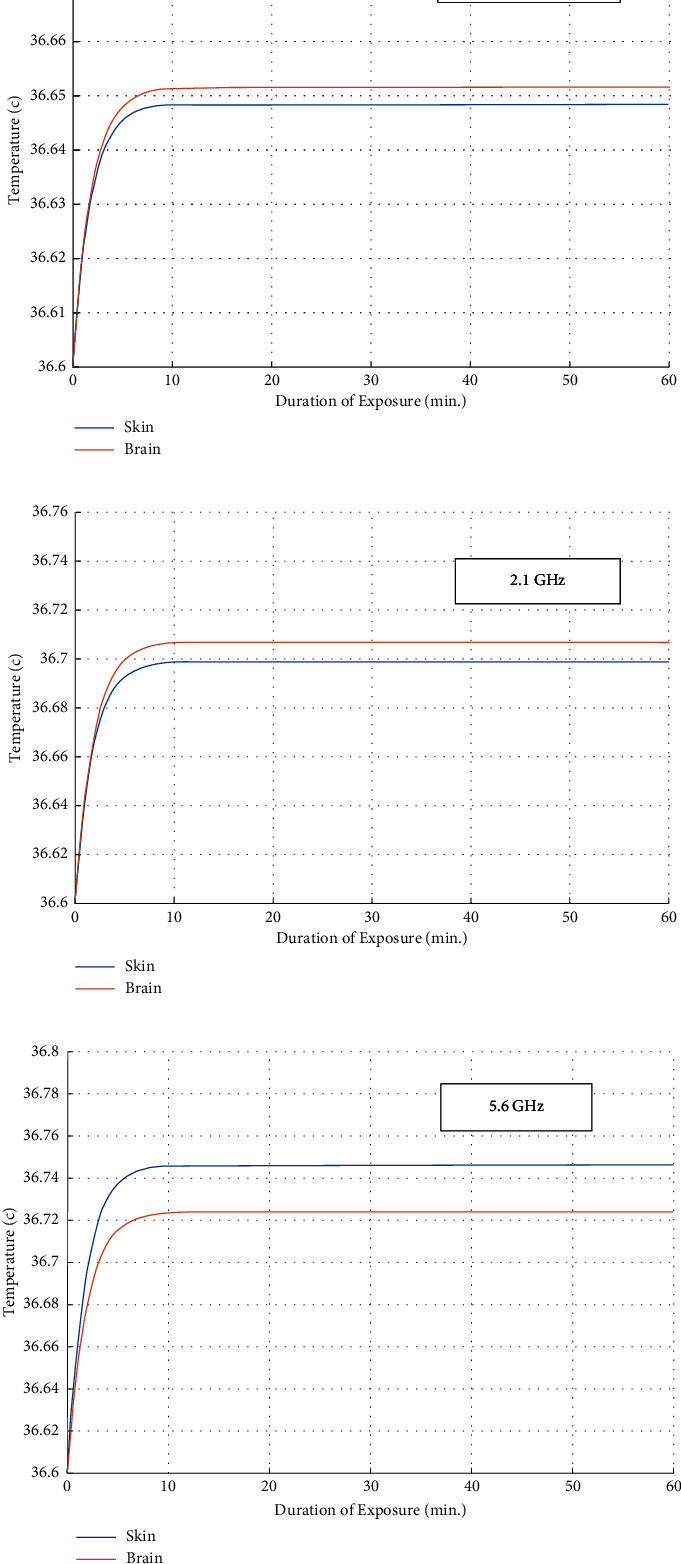
Effect of exposure time on tissues in terms of temperature, when *f* is (a) 850 MHz, (b) 2.1 GHz, and (c) 5.6 GHz.

Figure 4 shows the relationship between temperature and SAR on the tissues of skin and brain when the frequency is 850 MHz, 2.1 GHz, and 5.6 GHz, input power is 120 mW, at 0 distance between human's body and antenna.

The effect of exposure time on human tissues in terms of temperature depends on the duration of exposure to a heat source or energy, which is the radiofrequency radiation; the longer the exposure time, the more opportunity there is for heat to accumulate in the tissues.

The higher frequency, the higher heat accumulates in the tissues, which raises critical questions about the safety of 5 G and other technologies radiation.

In this study, the amount of peak power is considered as for the largest cells of 5 G networks, while the actual transmitted power is 250 mW for a small cell. And so, increasing the transmitted power will generate more heat based on equation (9) and as shown in Figure 3.

### 8.1. The Mechanism of 5 G Effect on Human Health

One mechanism by which 5 G technology might affect human health involves the generation of ROS and subsequent oxidative stress [23] [50–52].

When the body is exposed to RF-EMF from 5 G technology, it can lead to the generation of ROS within cells. ROS are highly reactive molecules containing oxygen, such as superoxide radicals (O_2_·−), hydroxyl radicals (·OH), and hydrogen peroxide (H_2_O_2_). An imbalance between the production of ROS and the body's antioxidant defenses can lead to oxidative stress. Oxidative stress occurs when there is an excess of ROS, which can damage cellular components such as lipids, proteins, and DNA. This damage can disrupt cellular function and contribute to the development of various health conditions.

Oxidative stress induced by RF-EMF exposure may affect cellular processes in several ways. ROS can directly damage DNA, leading to mutations and genetic instability. This damage may increase the risk of cancer and other diseases. ROS can modify proteins, impairing their function and disrupting cellular processes such as enzyme activity and signal transduction. ROS can initiate lipid peroxidation, a process that damages cell membranes and alters membrane properties, affecting cell viability and integrity. ROS can target mitochondria, the cellular organelles responsible for energy production. Oxidative damage to mitochondria can impair their function, leading to decreased energy production and cellular dysfunction.

## 9. Conclusion

5 G technology has several effects on all aspects either positively or negatively. This work provided comprehensive details about 5 G and its impact on climate, air, and humans. It is clear that radio waves have an effect on the human body, who can afford upper limited power with specific value at specific frequency, and that power should not exceed the agreed value by international bodies in order to avoid any health risk.

This work highlighted the issue of high frequency radiations by modern technology and its effect on human's tissues. It shows that the higher frequency, the higher effect of human's tissue in terms of heating.

The consensus among experts is that 5 G mobile technology is not likely to pose a significant risk to human health when deployed within established safety guidelines. However, the scientific community should continue to conduct rigorous research to ensure the ongoing safety of this technology.

## Figures and Tables

**Table 1:** Mobile generation summary [2].

Aspect	1 G	2 G	3 G	4 G	5 G
Year introduced	1980s	Late 1980s	Early 2000s	2009 (commercial)	2019 (commercial)
Data transfer speed	Low (voice only)	Up to 64 kbps	Up to 384 kbps	Up to 100 mbps	Up to multi-Gbps
Technology	Analog	Digital (GSM)	Digital (CDMA)	LTE (long term evolution, OFDMA)	NR (new radio)—NOMA
Frequency bands	N/A	Various	Various	Broadband LTE bands	Broadband mmWave and Sub-6 GHz bands
Spectrum efficiency	N/A	Low	Medium	High	Very high
Latency	N/A	High	Moderate	Low	Ultra-low
Use cases	Voice calls	Voice calls, SMS	Voice calls, data	Data, streaming	Data, IoT, AR/VR, ultra-reliable low-latency communication (URLLC)
Connection density	N/A	Low	Moderate	High	Very high
Network architecture	Circuit-switched	Circuit-switched	Circuit-switched	Packet-switched	Packet-switched
Key technologies/Features	N/A	SMS	Multimedia messaging, mobile internet, video calls	LTE-advanced, carrier aggregation, MIMO	Massive MIMO, beamforming, network slicing

**Table 2:** Literature review comparisons.

Ref	Positive	Limitations
Belyaev [14]	Providing an overview of the complex dependence of the nonthermal microwave effects on various physical and biological variables	Neither simulation nor tabular summary were provided
Mandl et al. [15]	Presenting two different measurement setups to study the effect of electromagnetic radiation	Relation between SAR and distance is not included
Fernández et al. [16]	Presenting an evaluation of electromagnetic exposure due to 5 G mobile communications in outdoor environments	Relation between SAR and distance is not included
Sofri et al. [17]	Presenting a review and analyses of the latest research on electromagnetic exposure on humans	No specific study or analysis has been done
Thotahewa et al. [18]	Presenting the electromagnetic effects of head implantable transmitting devices operating based on (IR-UWB), SAR), SA and temperature increase are analysed	Nonthermal effect did not be considered
Alcaras and Frere [19]	Presenting new concept of heterogeneous phantom to enhance SAR method validation of radio military exposure near high power antennas	Nonthermal effect did not be considered
Laissaoui et al. [20]	Presenting dosimetrist study by calculating the SAR and the temperature in the human head exposed to electromagnetic radiation emitted by a smartphone	It focused on human's head; other types of tissues were not considered
Nasiar and Rani [21]	Discussing the dangers of the mobile phone radiations on human health. Studied the effect of mobile phone on sleeping pattern, distance from the base tower, ill effect on health, average usage of Internet, and getting stressed with these devices	No study of thermal effect has been done
Annalakshmi and Umarani [22]	Investigating the human EMF exposure from 5 G wireless communication. It analyses the impact of the penetration level of EMF in human skin at 28 GHz	It considered skin tissues only; other types of tissues were not considered
Di Ciaula [23]	Providing comprehensive review about the effect of radio waves on human's tissues	No practical results nor simulation was provided
Simkó and Mattsson [24]	Analysing 94 relevant publications performing in vivo or in vitro investigations	No practical results nor simulation was provided

**Table 3:** Dielectric properties of the tissues of human's body [47].

	Permittivity			Conductivity (S/m)		
Tissue type	850 MHz	2.1 GHz	5.6 GHz	850 MHz	2.1 GHz	5.6 GHz
Blood	61.523	58.851	52.894	1.516	2.261	6.2208
Brain	52.976	36.6	32.826	0.921	1.0466	3.3307
Skin	41.676	38.431	35.28	0.850	1.3075	3.5467

**Table 4:** Comparison between calculated SAR and SAR by FCC [48, 49].

	SAR (W/kg)			FCC SAR (W/kg)
Tissue type	850 MHz	2.1 GHz	5.6 GHz	
Blood	0.860	1.586	52.656	1.6
Brain	0.249	0.648	30.562	1.6
Skin	0.263	1.102	17.008	1.6

## Data Availability

Data used to support the findings of this study are included within the article.
